# Facile Green Synthesis of N-Type InP Thin-Film Photoanodes with Enhanced Photoelectrochemical Performance for Solar Hydrogen Generation

**DOI:** 10.3390/nano15201544

**Published:** 2025-10-10

**Authors:** Ying-Chu Chen, Heng-Yi Lin, Yu-Kuei Hsu

**Affiliations:** 1 Department of Chemical Engineering & Biotechnology, National Taipei University of Technology, Taipei City 10608, Taiwan; 2 Department of Opto-Electronic Engineering, National Dong Hwa University, No. 1, Section 2, Da Hsueh Road, Hualien 97401, Taiwan

**Keywords:** indium phosphide, thin film, tin dopant, photoelectrochemistry

## Abstract

Indium phosphide (InP) is a promising photoactive material for solar-driven hydrogen production owing to its optimal bandgap, high carrier mobility, and broad solar absorption. However, conventional InP fabrication relies on costly wafers and toxic precursors, limiting its scalability and sustainability. Here, we demonstrate a simple and environmentally friendly route to synthesize n-type InP thin-film photoanodes by phosphidating indium films prepared via doctor blade coating on ITO substrates, using NaH_2_PO_2_ as a phosphorus source. Structural and spectroscopic analyses (XRD, Raman, XPS, PL) confirmed the successful formation of crystalline InP with optimum quality at 425 °C. Photoelectrochemical measurements revealed a significant photocurrent density of 1.8 mA·cm^−2^ under AM 1.5 illumination, with extended photoresponse into the near-infrared region. Mott–Schottky and EIS analyses indicated efficient charge separation, low transfer resistance, and unintentional n-type doping due to Sn diffusion from the ITO substrate. This facile and green synthesis route not only provides a scalable approach to III–V semiconductors but also highlights InP thin films as cost-effective and efficient photoanodes for sustainable solar hydrogen generation.

## 1. Introduction

Photoelectrochemical (PEC) water splitting has emerged as a highly promising strategy for converting solar energy into clean and renewable hydrogen fuel. Since the pioneering report of PEC hydrogen generation using TiO_2_ photoelectrodes, significant research efforts have been directed toward enhancing light-to-hydrogen conversion efficiency [1,2,3]. A key challenge lies in the development of efficient photoanodes, which require higher energy input for the oxygen evolution reaction. Various semiconducting materials, such as ZnO, WO_3_, and Fe_2_O_3_, have been investigated to improve photoanode performance [4,5,6]. However, TiO_2_ and ZnO are limited by their wide bandgaps, restricting absorption to the ultraviolet region, while Fe_2_O_3_ suffers from a low absorption coefficient and short carrier diffusion length. These intrinsic drawbacks result in low solar-to-hydrogen (STH) conversion efficiencies, underscoring the urgent need for alternative photoactive materials. Indium phosphide (InP) is considered one of the most promising candidates for PEC hydrogen generation due to its favorable direct bandgap of ~1.35 eV, which is well aligned with the solar spectrum, together with its high carrier mobility and low surface recombination velocity [7,8]. In the past decades, p-type InP photocathodes have been extensively studied, achieving remarkable efficiencies, with recent reports demonstrating power-saved efficiencies up to 15.8% at 0.65 V [9]. Despite these advances, studies on InP-based photoanodes remain relatively scarce. Most reported InP electrodes, whether nanostructured or thin-film tandem designs, rely on commercially available InP wafers that are both energy-intensive and costly to fabricate [10,11]. Furthermore, conventional InP growth techniques often require hazardous indium and phosphide precursors, raising concerns regarding environmental safety and scalability. To address these challenges, it is highly desirable to develop a facile, sustainable, and cost-effective strategy for preparing n-type InP photoanodes suitable for PEC water splitting. In this work, we propose a simple green synthesis route in which indium films are first deposited onto indium tin oxide (ITO) substrates through a doctor blade method, followed by phosphidation via thermal decomposition of NaH_2_PO_2_. This approach not only eliminates the need for expensive single-crystal wafers and toxic precursors, but also enables scalable fabrication of III–V phosphide semiconductors under relatively mild conditions.

In this study, the structural, optical, and photoelectrochemical properties of the synthesized InP films were systematically investigated. Particular attention was paid to the effect of phosphidation temperature on crystal quality, bandgap characteristics, and PEC performance. Remarkably, the optimized InP film exhibits strong photocurrent response, extended spectral activity into the near-infrared region, and efficient charge transport behavior. Beyond PEC hydrogen production, this facile green process also provides new opportunities for the design and large-scale fabrication of III–V semiconductors for broader optoelectronic and energy-related applications.

## 2. Experiments

### 2.1. Chemicals

Metallic indium (In, 99%), sodium phosphinate (NaH_2_PO_2_, 99%), and sodium hypophosphite (NaH_2_PO_2_, 98%) were obtained from Sigma-Aldrich. ITO was purchased from Ruilong Optoelectronics Co., Ltd. (Miaoli County, Taiwan). Hydrogen chloride (HCl, 30–50%), sodium sulfide (Na_2_S, 98%) and sodium sulfite (Na_2_SO_3_, 98%) were purchased from Fisher Scientific. All chemicals were of analytical grade and used as received without further purification.

### 2.2. Preparation of InP Thin Films

**Scheme 1:**
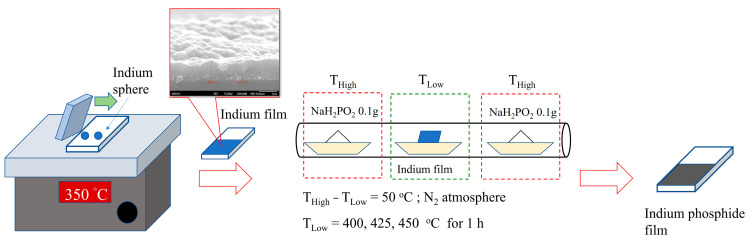
Schematic diagram of InP thin film synthesis.

The InP thin films were synthesized on indium tin oxide (ITO) substrates via a two-step process: (i) doctor blade deposition of metallic indium, followed by (ii) phosphidation treatment using NaH_2_PO_2_ as the phosphorus source (Scheme 1). First, ITO substrates were sequentially cleaned with ethanol, acetone, HCl, and deionized water, and then dried under nitrogen flow. Metallic indium spheres were preheated and subsequently spread onto the hot ITO substrate (350 °C) by doctor-blading to form a uniform indium layer. The thickness of the indium precursor film was measured to be approximately 900 nm by cross-section SEM. This thickness was selected after several preliminary trials to ensure complete phosphidation while avoiding peeling or discontinuity in the resulting InP film. The constancy and uniformity of the In layer were achieved by carefully controlling the doctor blade coating process at a fixed temperature (350 °C) and using the same amount of indium source. For phosphidation, an indium-coated ITO substrate was placed in the center of the furnace, while the other two alumina crucibles at both ends served only as containers for 0.1 g of NaH_2_PO_2_ to generate PH_3_ vapor. After purging with N_2_, the furnace center zone was heated to 400, 425, or 450 °C and maintained for 1 h, while the two end zones were heated to 450, 475, and 500 °C, respectively. During the reaction, NaH_2_PO_2_ decomposed to generate PH_3_ gas, which reacted with metallic indium to form InP. The system was naturally cooled to room temperature after completion.

### 2.3. Characterization

The morphology of the films was examined by field-emission scanning electron microscopy (SEM, JEM-4000EX, JEOL, Tokyo, Japan). Crystal structure was analyzed using X-ray diffraction (XRD, Bruker D8 Advance, Karlsruhe, Germany, Cu Kα radiation, λ = 0.1506 nm). Chemical composition and bonding states were characterized by X-ray photoelectron spectroscopy (XPS, Perkin-Elmer PHI 1600, Shelton, CT, USA) and Raman spectroscopy (LabRAM HR 550, HORIBA, Palaiseau, France, He–Ne laser, 632.8 nm, 5 mW). Photoluminescence (PL) spectra were recorded with a diode laser excitation source (405 nm, 50 mW) on a LabRAM HR 320 system (HORIBA, Palaiseau, France).

### 2.4. Photoelectrochemical Measurements

PEC performance was evaluated in a three-electrode configuration with the InP film as the working electrode, a platinum sheet as the counter electrode, and an Ag/AgCl (3 M KCl) electrode as the reference. The electrolyte consisted of 0.35 M Na_2_S and 0.5 M Na_2_SO_3_ aqueous solution. All potentials reported were converted to the reversible hydrogen electrode (RHE) scale (EAg/AgCl = 0.207 V vs. RHE). Illumination was provided by a 150 W Xe lamp equipped with an AM 1.5 filter, calibrated to 100 mW·cm^−2^ at the sample surface. Monochromatic incident photon-to-current efficiency (IPCE) spectra were obtained using a Xe lamp coupled with a monochromator. The incident light was directed onto the film surface through a quartz window and electrolyte. Electrochemical impedance spectroscopy (EIS) was performed at 0.8 V vs. RHE under illumination using a frequency range of 100 kHz to 0.1 Hz with an AC amplitude of 10 mV at varied applied potentials. Linear sweep voltammetry (LSV) under chopped illumination was conducted at a scan rate of 10 mV·s^−1^ to record photocurrent responses.

## 3. Results and Discussion

### 3.1. Characterization of Structure and Composition

**Figure 1:**
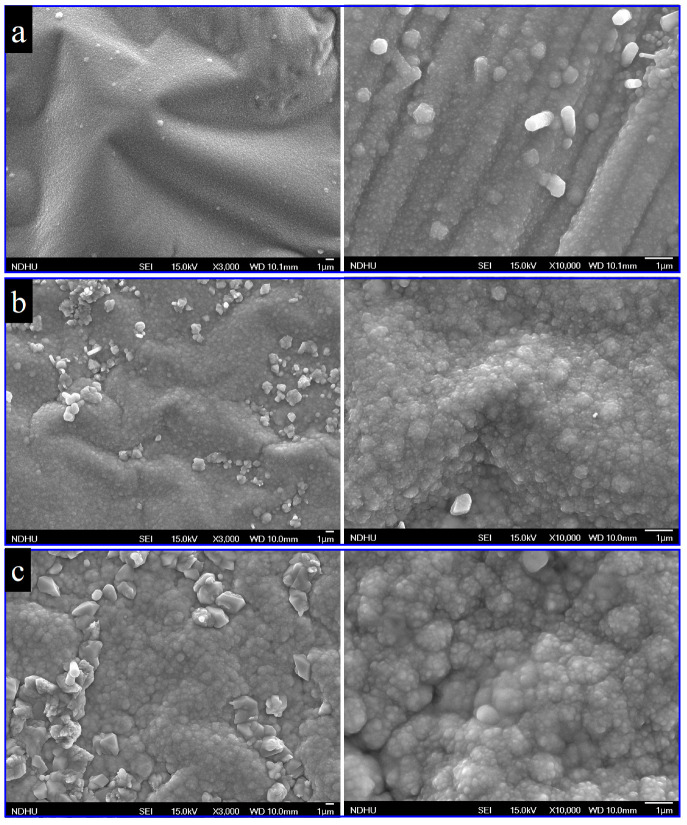
FE-SEM images of InP films at the growth temperature of (**a**) 400 °C, (**b**) 425 °C and (**c**) 450 °C.

**Figure 2:**
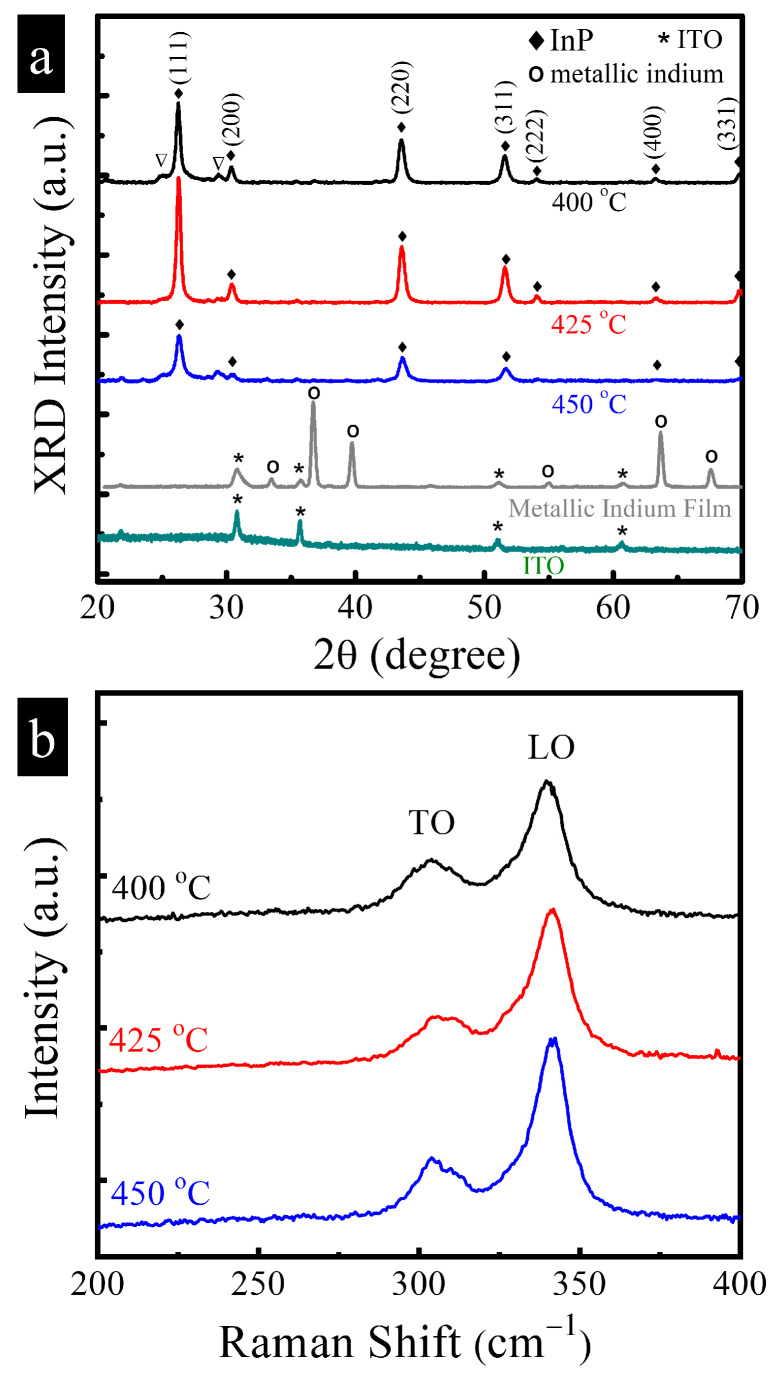
(**a**) X-ray diffraction pattern and (**b**) Raman spectra of InP films.

The morphology of InP films prepared at different phosphidation temperatures is shown in Figure 1. At 400 °C, the film exhibited a continuous and relatively smooth surface (Figure 1a). Increasing the temperature to 425 °C resulted in a slightly rougher surface with tightly connected clusters that still maintained a continuous film structure (Figure 1b). In contrast, at 450 °C, the clusters became more dispersed, leading to discontinuous and porous morphologies (Figure 1c). These observations indicate that temperature plays a critical role in film continuity and microstructural evolution. The crystal structures of the films were further analyzed by X-ray diffraction (XRD, Figure 2a). Prior to phosphidation, the diffraction peaks could be assigned to tetragonal metallic indium (JCPDS No. 05-0642) along with signals from the ITO substrate. After phosphidation, the dominant diffraction peaks at 2θ = ~25.3°, ~43.6° and ~51.5° (marked ◆ in Figure 2a) are indexed to the zinc-blende (ZB) phase of InP (JCPDS No. 32-0452), with the strongest (111) reflection located at ~25.3°. Two weaker features located on the shoulders of the main (111) peak, at ~24.6° and ~27.1° (marked ▽), can be assigned to contributions from wurtzite (WZ)-like stacking or polytypism in InP, consistent with previous reports that InP can display mixed ZB/WZ character under non-equilibrium growth conditions [12]. The absence of residual indium peaks confirms complete transformation of metallic indium into InP via PH_3_ generated from NaH_2_PO_2_ decomposition [13]. The Scherrer analysis of the (111) diffraction peaks revealed grain sizes of 18.9, 20.4, and 13.9 nm for films grown at 400, 425, and 450 °C, respectively. The (111) peak intensity followed the order 425 °C > 400 °C > 450 °C, indicating that the best crystallinity was achieved at 425 °C. Raman spectroscopy provided additional evidence of the crystalline quality (Figure 2b). All samples exhibited a strong longitudinal optical (LO) phonon mode at 340 cm^−1^ and a weaker transverse optical (TO) mode at 305 cm^−1^, characteristic of InP [14]. The LO/TO intensity ratio was highest for the 425 °C sample, further confirming its superior crystal quality, consistent with the XRD results.

**Figure 3:**
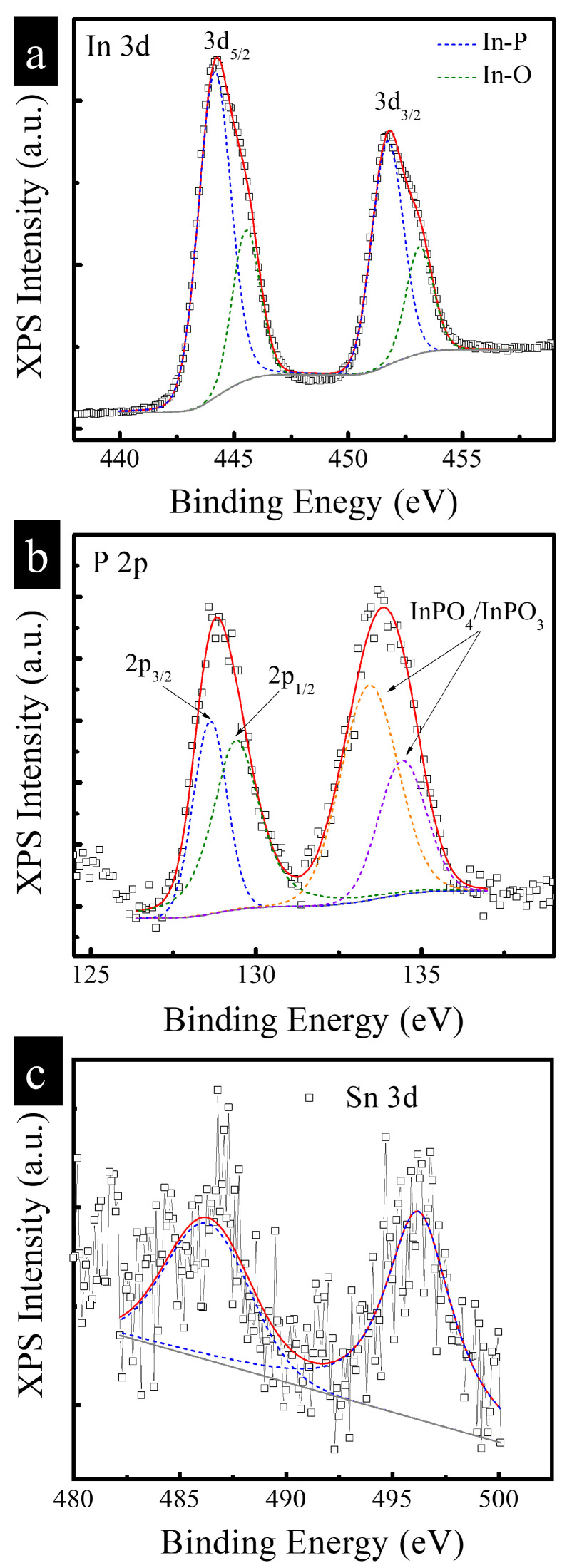
(**a**) In 3d, (**b**) P 2p and (**c**) Sn 3d XPS spectra of InP film at 425 °C.

**Figure 4:**
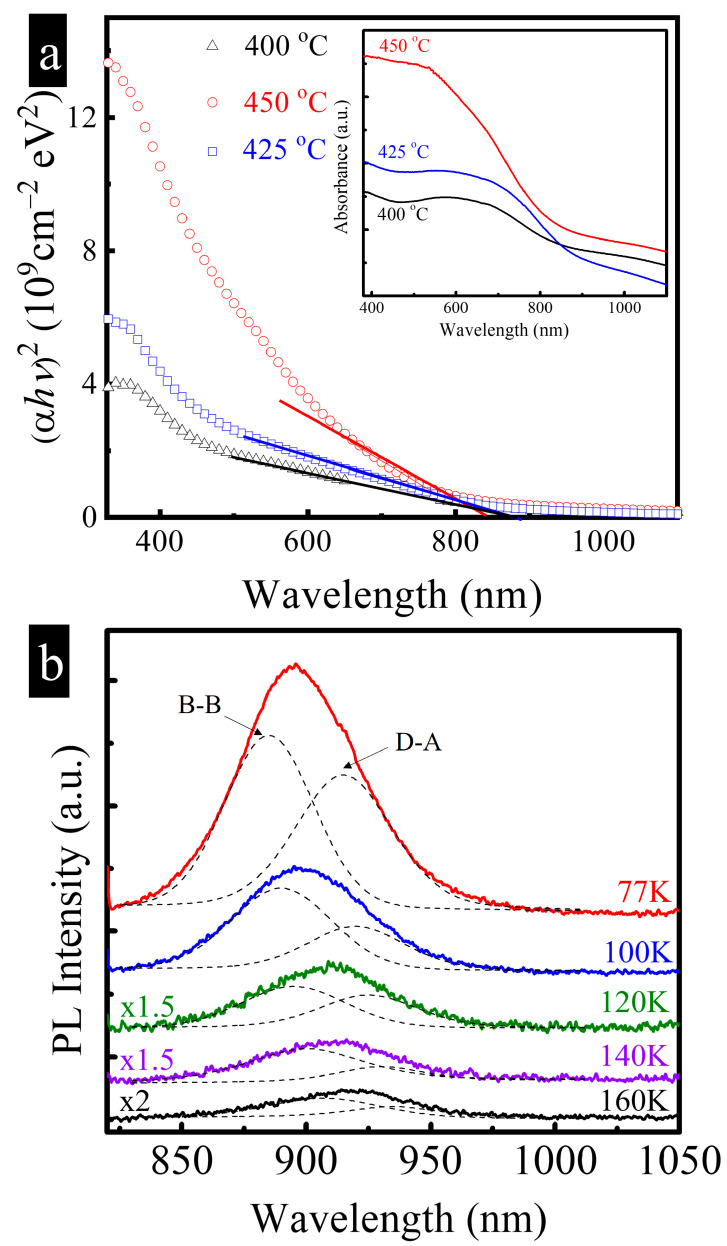
(**a**) Absorbance spectra and (**b**) temperature-dependent PL spectra of InP films.

The chemical composition was analyzed by X-ray photoelectron spectroscopy (XPS, Figure 3). For the film prepared at 425 °C, the In 3d doublet at 444.2 and 451.9 eV was assigned to In–P bonding, with additional minor peaks from indium oxide species, likely due to surface oxidation. The P 2p spectrum displayed characteristic doublets at 128.6 and 129.4 eV corresponding to P^3−^ in InP, together with a higher binding energy component (~134 eV) indicative of oxidized phosphorus species (InPO_3_/InPO_4_) [15]. The In/P atomic ratio was approximately 1.15:1, consistent with near-stoichiometric InP formation. Notably, a small amount of Sn^4+^ (0.6 at.%) was detected, likely due to out-diffusion from the ITO substrate, suggesting unintentional n-type doping of the InP films. Optical absorption measurements (Figure 4a) revealed that all films displayed absorption edges around 900 nm, consistent with InP band-to-band transitions. The optical bandgaps were estimated as 1.43, 1.49, and 1.44 eV for films grown at 400, 425, and 450 °C, respectively, aligning well with literature values [9,10,11]. Low-temperature photoluminescence (PL) spectra of the 425 °C film (Figure 4b) the peak exhibits an asymmetric broadening, which we initially fitted into two components: a band-to-band (B−B) transition at 1.401 eV and a donor–acceptor (D−A) pair recombination at 1.355 eV. Both peaks redshifted with increasing temperature, primarily due to electron–phonon interactions and, to a lesser extent, thermal lattice expansion [16]. Taken together, the structural, compositional, and optical analyses demonstrate that phosphidation at 425 °C produces InP films with the best crystallinity, near-stoichiometric composition, and favorable electronic transitions, providing a strong foundation for enhanced PEC performance.

### 3.2. PEC Performance of InP Film Photoanodes

**Figure 5:**
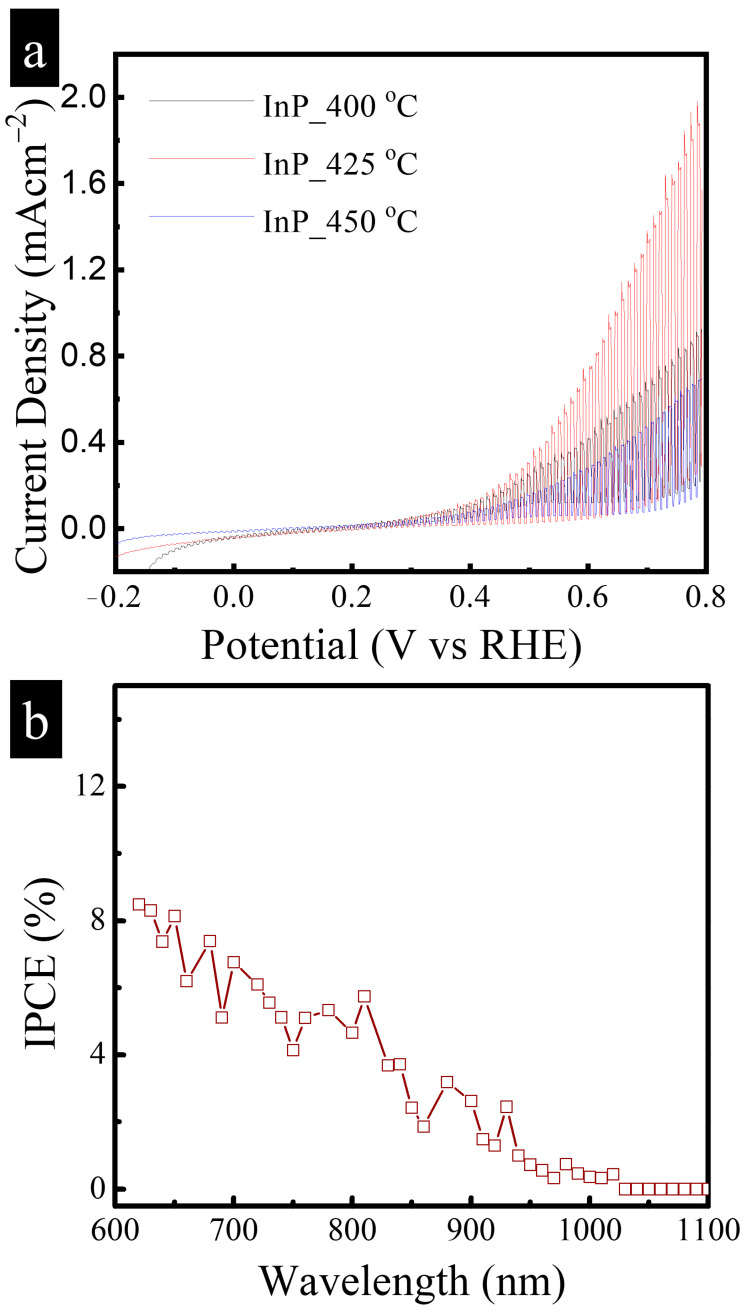
(**a**) Photocurrent response and (**b**) IPCE as a function of excitation wavelength of the InP films.

**Figure 6:**
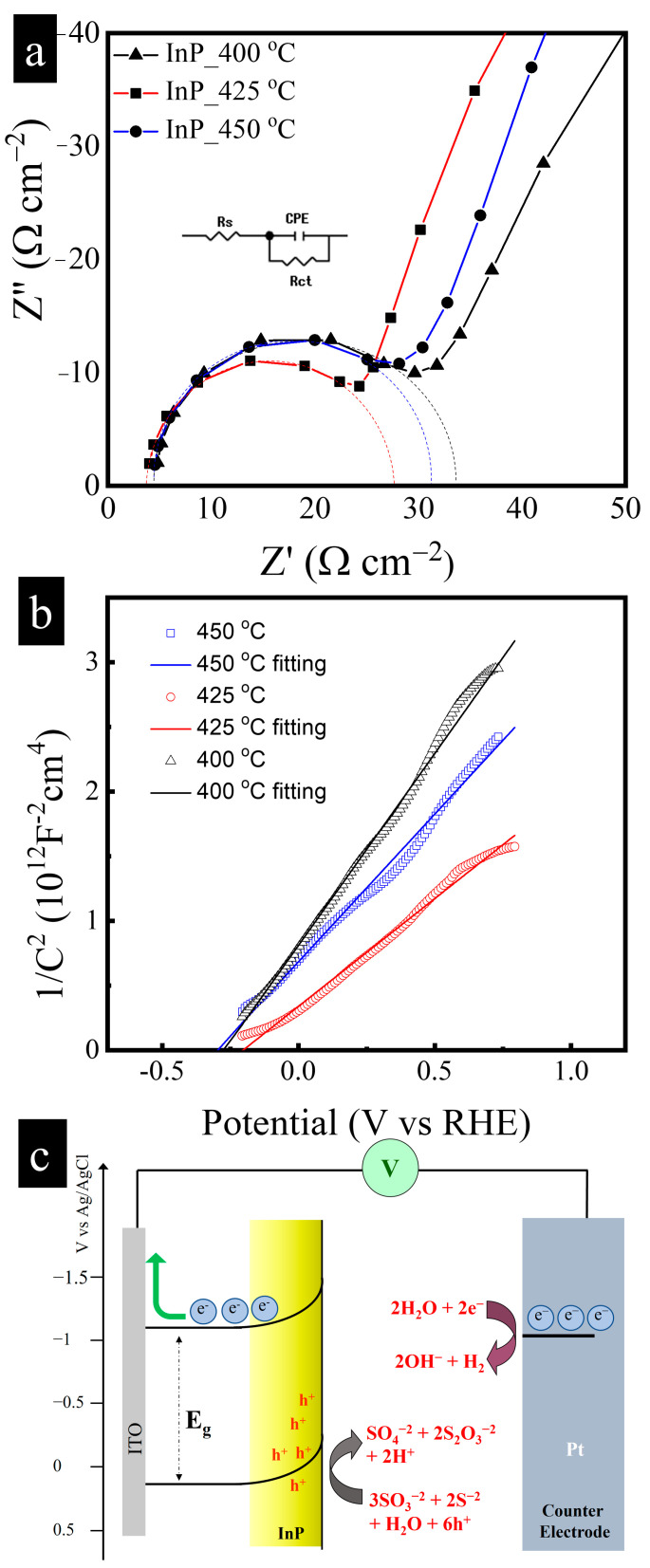
(**a**) Nyquist plots and (**b**) Mott–Schottky analysis of InP films; (**c**) Energy diagram of InP photoelectrode.

The photoelectrochemical behavior of the InP thin-film electrodes was evaluated in a three-electrode cell using a mixed sulfide/sulfite electrolyte (0.35 M Na_2_S + 0.5 M Na_2_SO_3_) as a sacrificial hole scavenger. It should be noted that the Na_2_S/Na_2_SO_3_ electrolyte system was employed in this study as a sacrificial medium, where Na_2_SO_3_ acts as a hole scavenger to facilitate surface redox reactions and suppress photocorrosion of the InP photoanodes. While this configuration allows the intrinsic photoelectrochemical activity and interfacial charge-transfer kinetics to be evaluated under stable operation, it does not represent the actual oxygen evolution process in pure water. The use of Na_2_SO_3_ thus highlights the current limitation of bare InP photoanodes, which are prone to surface oxidation at more positive potentials. Future efforts will focus on improving stability and enabling oxygen evolution through the integration of surface passivation layers, protective coatings, or catalytic over-layers. Linear sweep voltammetry under chopped illumination (Figure 5a) revealed a clear anodic photocurrent response for all samples, confirming the n-type conductivity of the InP films. This unintentional n-type character is likely attributed to the diffusion of Sn from the ITO substrate during phosphidation. Among the three samples, the film prepared at 425 °C exhibited the highest photocurrent density, reaching 1.8 mA·cm^−2^, which can be correlated with its superior crystallinity evidenced by XRD and Raman analyses. To further evaluate the PEC performance of our InP photoanodes, it is instructive to compare them with other indium-based photoanode materials. In_2_S_3_ has been widely studied as a visible-light-responsive semiconductor; however, its photocurrent density is usually very low, typically ~1.3 mA·cm^−2^, even when modified with 2D MoS_2_ nanosheets to improve charge separation [17]. Similarly, In_2_O_3_ photoanodes have demonstrated improved stability but limited photocurrent response, with a maximum photocurrent density of only 0.97 mA·cm^−2^ under simulated solar illumination [18]. In contrast, our n-type InP thin-film photoanode achieves a photocurrent density of 1.8 mA·cm^−2^ without additional cocatalyst or protective layers. This performance not only surpasses that of other indium-based photoanodes, but also underscores the intrinsic advantages of InP, including its optimal direct bandgap and high carrier mobility. These results suggest that InP, prepared via our facile and green phosphidation method, offers a promising pathway toward efficient and scalable PEC water splitting devices. The spectral response of the electrodes was further examined by incident photon-to-current efficiency (IPCE) measurements, which were performed at 0.8 V vs. RHE (Figure 5b). The 425 °C sample displayed a broad photoresponse spanning the visible to near-infrared region, with an onset wavelength of ~900 nm, consistent with the bandgap of InP. This extended photoresponse highlights the capability of InP films to harvest a wide range of the solar spectrum for hydrogen production. In addition, electrochemical impedance spectroscopy (EIS) was employed to investigate interfacial charge transfer under illumination (Figure 6a). To extract interfacial kinetic parameters, the Nyquist data were fit using a simple equivalent circuit consisting of the series resistance (R_s_) in series with a parallel combination of charge transfer resistance (R_ct_) and a constant phase element (CPE) [19]. The R_ct_ value for the 425 °C sample is significantly smaller than for the 400 °C and 450 °C samples (fitting values: R_ct_ (425 °C) = 28.5 Ω cm^2^, R_ct_ (400 °C) = 33.7 Ω cm^2^, R_ct_ (450 °C) = 31.4 Ω cm^2^), indicating faster interfacial charge transfer kinetics and improved charge extraction under illumination. A reduced R_ct_ is consistent with the observed higher photocurrent and is attributable to improved crystallinity and lower trap density at the semiconductor/electrolyte interface.

To gain further insight into the electronic structure, Mott–Schottky measurements were performed in the dark (Figure 6b). All samples exhibited positive slopes, confirming their n-type nature. To further extract the position of the flat-band potential and the surface carrier concentration of those materials, we utilized Mott-Schottky equation that relates the capacitance of a semiconductor to the carrier concentration (*N_d_*) and to other constants and parameters, such as the fundamental electric charge *e*, relative permittivity (*ε* of InP is 10.8 [10]), permittivity of vacuum (*ε*_o_), temperature (*T*), Boltzmann constant (*k_B_*), and the flat-band potential (*V_FB_*).

(1) $$\frac{1}{C^{2}}=(\frac{2}{e\varepsilon \varepsilon_{0}N_{d}})[V-V_{FB}-\frac{k_{B}T}{e}]$$

Plotting 1/*C*^2^ versus *V* allows the estimation of the flat-band potential and the concentration of surface carriers, the flat-band potential calculated from the abscissal intercept and the carrier concentration from the slope. The flat-band potential was determined to be approximately −0.3 V (vs. RHE), while the carrier concentrations were estimated in the range of 4 × 10^18^ to 7 × 10^18^ cm^−3^. The flat-band potential of InP film is also comparable or even more negative than those of In_2_S_3_ and In_2_O_3_, which typically range from 0.2 to −0.2 V vs. RHE [17,18]. These comparisons emphasize the superior PEC characteristics of InP and demonstrate its promising potential as a photoanode material. Based on these parameters and the measured bandgap, the energy band alignment was constructed (Figure 6c). Under illumination, photoexcited electrons in the conduction band are efficiently driven to the counter electrode for hydrogen evolution, while photogenerated holes migrate toward the electrolyte interface, where they are rapidly consumed by the sulfide/sulfite species. Taken together, these results demonstrate that InP films synthesized via the two-step process at 425 °C possess excellent PEC activity, enhanced photocurrent response, and improved charge transport properties. The favorable band alignment, high crystallinity, and efficient charge separation collectively enable their promising application as n-type photoanodes for solar hydrogen generation.

## 4. Conclusions

In this work, n-type indium phosphide (InP) thin films were successfully synthesized on ITO substrates via a simple two-step process involving doctor blade deposition of metallic indium followed by phosphidation using NaH_2_PO_2_ as the phosphorus source. Structural and compositional analyses confirmed the complete conversion of metallic indium into crystalline InP, with the optimized film obtained at a phosphidation temperature of 425 °C. This sample exhibited superior crystallinity, near-stoichiometric composition, and minimal structural defects, as evidenced by XRD, Raman, and XPS results. The optimized InP films displayed a direct bandgap of ~1.4 eV and extended optical absorption up to ~900 nm, enabling efficient utilization of both visible and near-infrared light. Photoelectrochemical measurements demonstrated excellent PEC activity, with a photocurrent density of 1.8 mA·cm^−2^ under AM 1.5 illumination. Enhanced charge separation and reduced interfacial resistance, confirmed by EIS and Mott–Schottky analyses, were attributed to improved crystallinity and unintentional Sn doping diffused from the ITO substrate. Overall, this study establishes a facile, low-cost, and environmentally friendly approach for fabricating InP thin-film photoanodes. The demonstrated method not only eliminates the need for expensive single-crystal wafers and toxic precursors, but also provides a scalable route to III–V semiconductors with excellent PEC performance. Beyond solar hydrogen generation, this green synthesis strategy offers new opportunities for the design of sustainable photoelectrodes and optoelectronic devices.

## Data Availability

The data are contained within the article.
